# The relationship between controlled ovarian stimulation protocol, meiotic spindle visibility, position of the meiotic spindle relative to the polar body in the human oocyte, and clinical outcomes following ICSI

**DOI:** 10.1002/rmb2.12601

**Published:** 2024-12-13

**Authors:** Taketo Inoue, Yuki Matsuo, Sayumi Taguchi, Yoshiko Tsujimoto, Mikiko Uemura, Yoshiki Yamashita

**Affiliations:** ^1^ Umeda Fertility Clinic Osaka Japan; ^2^ Department of Emergency, Disaster and Critical Care Medicine Hyogo Medical University Nishinomiya Hyogo Japan; ^3^ Department of Rehabilitation Science Kobe University Graduate School of Health Sciences Kobe Hyogo Japan; ^4^ Department of Rehabilitation, Faculty of Health Science Kansai University of Welfare Sciences Osaka Japan

**Keywords:** controlled ovarian stimulation, female age, intracytoplasmic sperm injection, spindle, visibility

## Abstract

**Purpose:**

To investigate the effects of different controlled ovarian stimulation (COS) protocols, including the progestin‐primed ovarian stimulation (PPOS), long, short, and the gonadotropin‐releasing hormone antagonist protocols, on meiotic spindle visibility and position within the oocyte and clinical outcomes following ICSI.

**Methods:**

Before ICSI, spindle position (*θ*) just below the polar body (PB) was defined as 0° and categorized as follows: *θ* = 0°, 0° < *θ* ≤ 30°, 30° < *θ* ≤ 60°, 60° < *θ* ≤ 90°, 90° < *θ* ≤ 180°, between the PB and the oolemma, and nonvisible. The clinical outcomes after ICSI were retrospectively analyzed.

**Results:**

The normal fertilization rate was significantly higher in oocytes with visible spindles than in oocytes with nonvisible spindles after each COS protocol, but did not differ based on spindle positioning (0° ≤ *θ* ≤ 180°). The rates of pregnancy, live birth/ongoing pregnancy, and miscarriage did not differ based on spindle visibility or positioning. In multinominal logistic regression analysis, female age was associated with spindle position, and the odds of a spindle located at 30° < *θ* ≤ 60°, at 60° < *θ* ≤ 90°, or at 90° < *θ* ≤ 180° were increased relative to *θ* = 0° in older women (odds ratio; 1.020, 1.030, and 1.060, respectively; *p* < 0.05).

**Conclusion:**

Meiotic spindle positioning in the oocyte does not affect normal fertilization, blastulation, pregnancy, live birth/ongoing pregnancy, and miscarriage after ICSI, independent of the COS protocol used.

## Abstract 2

The visibility of the meiotic spindle in oocytes impacted the normal fertilization rate, independent of controlled ovarian stimulation protocol. However, the spindle position within the ooplasm did not influence clinical outcomes, such as normal fertilization, blastulation, pregnancy, live birth, and miscarriage after controlled ovarian stimulation. Furthermore, female age did not affect spindle visibility; whereas the spindle position was associated with female age and moved away from directly beneath the polar body as female age increased.

## 1 INTRODUCTION

The meiotic spindle of a mature human oocyte can be located near the first polar body (PB), distal to the first PB, or may not be visible depending on the circumstances.^1^, ^2^ Several studies have investigated the relationships between visibility of the meiotic spindle and its position relative to the PB and normal fertilization and embryo development after intracytoplasmic sperm injection (ICSI).^1^, ^3^, ^4^ Previous studies demonstrated that the visibility of the meiotic spindle in the oocyte affects the rate of normal fertilization.^2^, ^3^, ^4^ The position of the meiotic spindle relative to the PB impacts normal fertilization, but little consensus has been reached regarding the effects of spindle visibility and position on embryogenesis.^1^, ^2^, ^3^

The relative positions of the meiotic spindle and PB are affected by several factors, including cytoplasmic maturation processes and the mechanical denudation procedure used before ICSI.^5^, ^6^, ^7^ Additionally, low ovarian reserve and excessive stimulation have been associated with a nonvisible spindle.^1^ During assisted reproduction, different controlled ovarian stimulation (COS) protocols that promote ovarian folliculogenesis may lead to different responses and endocrine environments for oocyte maturation.^8^ However, there are few reports on the relationship between the position of the spindle relative to the PB and fertilization/embryogenesis after different COS protocols. The gonadotropin‐releasing hormone (GnRH) agonist (long and short), GnRH antagonist, and progestin‐primed ovarian stimulation (PPOS) protocols have different effects on intraovarian autocrine and paracrine systems, as well as on the pituitary gland, to prevent a premature surge of luteinizing hormone during COS.^9^, ^10^ Thus, the GnRH agonist protocols suppress gonadotrophin secretion through both pituitary desensitization and GnRH receptor downregulation.^11^ In contrast, the GnRH antagonist protocol rapidly inhibits gonadotrophin secretion via a competitive blockade of GnRH receptors.^12^ In the PPOS protocol, progestin is used to suppress a premature luteinizing hormone surge during the follicular phase, thereby preventing premature ovulation.^13^ Accordingly, the potentially different effects of these COS protocols on ovarian folliculogenesis could lead to different responses and endocrine environments for maturing oocytes.^8^

To further explore these potential differences, we investigated the effects of different COS protocols on the spindle visibility and the position of the mature oocyte spindle, as well as the effects of spindle visibility and position on fertilization, embryonic development, pregnancy, and live birth after ICSI.

## 2 MATERIALS AND METHODS

### 2.1 Patients

The relationship between spindle position and COS protocol was retrospectively analyzed using data obtained from 1291 ICSI cycles from January 2020 through July 2023. ICSI cycles with vitrified‐thawed oocytes were excluded from all analyses. ICSI cycles using frozen‐thawed spermatozoa, spermatozoa retrieved by testicular sperm extraction, or oocytes that were artificially activated with calcium ionophore treatment were excluded from the analyses of fertilization, embryo development, pregnancy, live birth/ongoing pregnancy (LB/OP), and miscarriage.

### 2.2 Controlled ovarian stimulation and oocyte retrieval

COS was performed using the long GnRH agonist, short GnRH agonist, GnRH antagonist, or PPOS protocol depending on the patient as described previously.^13^, ^14^, ^15^, ^16^ A GnRH analogue acetate (Fuji Pharma Co., Tokyo, Japan), human menopausal gonadotropin (ASKA Pharmaceutical Co., Tokyo, Japan), a GnRH antagonist (Cetrotide; Merck KGaA, Darmstadt, Germany), and/or chlormadinone acetate (Lutoral tablets; Fuji Pharma Co.) were administered as dictated in these established protocols. When at least 2 follicles reached 18–20 mm in diameter, human chorionic gonadotropin (hCG; Fuji Pharma Co.) was administered as part of the long, short, and GnRH antagonist protocols. In the PPOS protocol, hCG and GnRH agonists were administered. Oocyte retrieval was performed 34–36 h post‐hCG‐administration. The cumulus‐oocyte complexes were placed into 4‐well dishes (Thermo Fisher Scientific, Waltham, MA, USA) containing ORIGIO Sequential Fert (CooperSurgical, Ballerup, Denmark) and cultured until denudation.

### 2.3 Intracytoplasmic sperm injection and embryo culture

**FIGURE 1:**
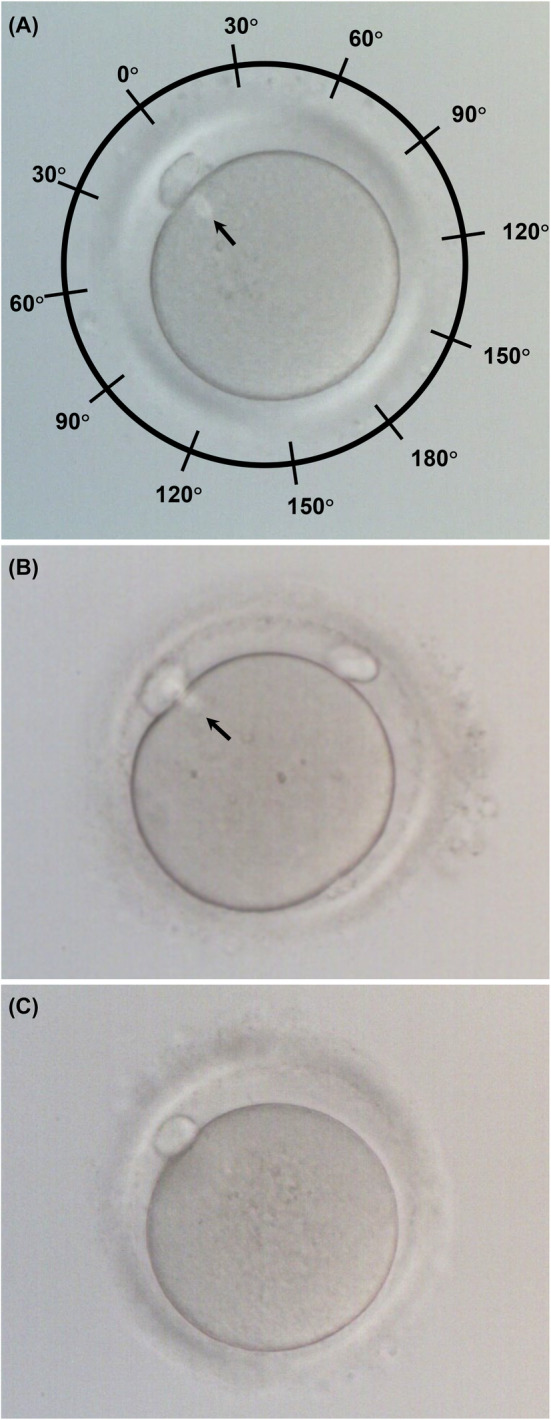
The positioning of meiotic spindle relative to the polar body in the human oocyte. (A) Oocyte with a visible spindle. The relative position of the spindle just below the polar body was defined as 0°. (B) Oocyte with the spindle between the PB and the oolemma. (C) Oocyte with a nonvisible spindle. Arrow: Meiotic spindle.

Oocytes were freed from cumulus cells using 80 IU/mL hyaluronidase solution (Fujifilm Irvine Scientific, Santa Ana, CA, USA) by pipetting for 20–60 s. Subsequently, the oocytes were transferred to a modified human tubal fluid medium (mHTF, Kitazato Corporation, Shizuoka, Japan) with HEPES (N‐2‐hydroxyethylpiperazine‐N2‐ethane sulfonic acid) containing 10% serum protein substitute (Kitazato Corporation). The remaining cumulus cells and corona radiata cells were then stripped from the oocytes by gradually decreasing the inner diameter of the pipettes used for manipulation (3–6 types were used). The time from hCG administration to completion of denudation was 37.4 ± 1.1 h (range 34.4–42.5 h). Following denudation, metaphase II (MII) oocytes were cultured before ICSI procedures. Immobilization of motile sperm was performed in a 5‐μL drop of 7% polyvinylpyrrolidone (PVP solution; Fujifilm Irvine Scientific). The relative positions of the meiotic spindle and PB were confirmed by visualization under polarization microscopy (IX73; Olympus, Tokyo, Japan) while rotating the oocyte in all directions using the injection and holding pipettes. When a weak light spindle was observed, it was classified as visible. Subsequently, oocytes were inseminated through ICSI. Time from hCG administration to the completion of ICSI (hCG‐ICSI interval) was 40.0 ± 1.6 h (range: 34.8–44.5 h). The relative position of the spindle just below the PB (*θ*) was defined as 0°, and spindle position was categorized as follows: *θ* = 0°, 0° < *θ* ≤ 30°, 30° < *θ* ≤ 60°, 60° < *θ* ≤ 90°, and 90° < *θ* ≤ 180°, PB/oolemma (between the PB and the oolemma),^17^, ^18^ and nonvisible (Figure 1). After ICSI, inseminated oocytes were cultured in an 80‐μL drop of SAGE 1‐Step media (CooperSurgical) covered with 4 mL of light mineral oil (Oil for Embryo Culture; Fujifilm Irvine Scientific) for 7 days (Geri+; Genea Biomedx, Sydney, NSW, Australia, CO_2_, 6%; O_2_, 5% at 37°C and 76.5% humidity).

### 2.4 Cryopreservation, warming, and blastocyst transfer

Blastocysts were vitrified and warmed using Cryotec (Reprolife, Tokyo, Japan) according to the manufacturer's instructions. Each patient received a single, vitrified‐warmed blastocyst, which was transferred into the uterus. Data were obtained from 786 vitrified‐warmed blastocyst transfer (VBT) cycles from January 2020 through July 2023. Luteal support for VBT cycles was performed as previously described.^19^ Following the initiation of menstruation, 0.72 mg transdermal estradiol patches (Estrana Tape; Hisamitsu Pharmaceutical Co., Tokyo, Japan) were applied to the abdomen for 10 weeks. Two 200 mg progesterone vaginal capsules (Utrogestan vaginal capsules 200 mg; Fuji Pharma. Co.) were administered twice per day when the endometrial thickness reached at least 7.0 mm. From the day of VBT, 125 mg of 17α‐hydroxyprogesterone caproate (progesterone depot intramuscular injection; Fuji Pharma. Co.) was injected every 5 days until 8 weeks of pregnancy. Pregnancy and ongoing pregnancy were identified by ultrasonographic monitoring of the gestational sac (GS) and fetus. A live birth was determined in accordance with the definitions of live birth provided by the World Health Organization.^20^

### 2.5 Statistical analysis

Data are presented as mean ± standard deviation. Normality was tested with Shapiro–Wilk normality tests. Female age and the hCG‐ICSI interval were considered as continuous variables and evaluated using the Steel–Dwass test. The rates of spindle visibility, fertilization, blastulation, biochemical pregnancy (hCG‐positive), clinical pregnancy (GS‐positive), fetal heart movement (FHM)‐positive, LB/OP, and miscarriage were evaluated using the Holm test. The relationships between spindle visibility and several factors, such as COS protocol, female age, and the hCG‐ICSI interval,^21^ were analyzed using univariate logistic analysis. The variance inflation factor (VIF) for each factor was <5 (COS protocol, 1.13; female age, 1.30; hCG‐ICSI interval, 1.32) indicating minimal multicollinearity, and a multiple logistic regression analysis was conducted accordingly. The relationships between the position of the spindle relative to the PB and several factors, including COS protocol, female age, and the hCG‐ICSI interval, were analyzed in the multinominal logistic regression analysis. The ratios of clinical outcomes between oocytes with a visible or nonvisible meiotic spindle were evaluated using Fisher's exact test. The statistical analysis was performed using EZR software,^22^ and a probability level of *p* < 0.05 was considered statistically significant.

## 3 RESULTS

### 3.1 The relationship between controlled ovarian stimulation protocol and clinical outcomes after intracytoplasmicsperm injection

In this retrospective analysis of the impact of different COS protocols on oocyte maturation, ICSI, and embryo development, the mean age of the women who provided oocytes was 38.2 ± 5.1 years when ovum pickup (OPU) was performed (range 23–50 years). Female patients treated with the GnRH antagonist protocol (39.8 ± 4.8 years) were significantly older when OPU was performed than patients treated with the PPOS (36.2 ± 5.1 years), long (34.6 ± 4.1 years), or short (36.6 ± 4.0 years) protocol (*p* < 0.01, Table 1). Furthermore, the female patients treated using the short protocol were significantly older when OPU was performed than patients treated using the long protocol (*p* < 0.01, Table 1). Nonetheless, the percentage of MII oocytes recovered using the PPOS (2383/2987, 79.8%) or short (1560/1954, 79.8%) protocols was significantly higher than that recovered using the long (434/605, 71.7%) or GnRH antagonist (2291/3225, 71.0%) protocols (*p* < 0.01, Table 1).

**TABLE 1:** The relationship between the stage of oocyte maturation, ICSI, or embryo development and controlled ovarian stimulation protocol.

Outcome	Controlled ovarian stimulation protocol			
	PPOS	Long	Short	GnRH antagonist
Oocyte maturation				
No. of cycles	311	55	229	696
Female (oocyte provider) age, mean years ± SD^a^	36.2 ± 5.1	34.6 ± 4.1	36.6 ± 4.0^§§^	39.8 ± 4.8**^,§§,††^
	*n* (%)	*n* (%)	*n* (%)	*n* (%)
Oocyte retrieval	2987	605	1954	3225
MII oocytes^b^	2383 (79.8)	434 (71.7)**	1560 (79.8)^§§^	2291 (71.0)**^,††^
MI oocytes^b^	283 (9.5)	73 (12.1)	149 (7.6)^§§^	352 (10.9)††
GV oocytes^b^	215 (7.2)	57 (9.4)	125 (6.4)**	329 (10.2)**^,††^
Broken/other oocytes^b^	106 (3.5)	41 (6.8)**	120 (6.1)**	253 (7.8)**
ICSI				
No. of ICSI cycles^c^	283	47	207	575
Female (oocyte provider) age, mean years ± SD^a^	36.1 ± 5.0	34.9 ± 4.1	36.5 ± 4.0	39.7 ± 4.8**^,§§,††^
	*n* (%)	*n* (%)	*n* (%)	*n* (%)
MII oocytes^b^	2166	367	1418	1943
2PN2PB oocytes^b^	1666 (76.9)	254 (69.2)*	1030 (72.6)**	1411 (72.6)**
1PN oocytes^b^	67 (3.1)	4 (1.1)	36 (2.5)	48 (2.5)
Multi‐PN oocytes^b^	59 (2.7)	14 (3.8)	60 (4.2)	90 (4.6)**
No‐PN oocytes^b^	94 (4.3)	25 (6.8)	75 (5.3)	100 (5.1)
Unfertilized oocytes^b^	174 (8.0)	53 (14.4)**	128 (9.0)^§§^	177 (9.1)^§§^
Broken oocytes^b^	106 (4.9)	17 (4.6)	89 (6.3)	117 (6.0)
Embryo development				
Cultured 2PN2PB^d^	1444	217	866	1062
Blastocysts^b^	997 (69.0)	142 (65.4)	557 (64.3)	665 (62.6)**
Pregnancy, live birth, and miscarriage				
Blastocyst transfer cycles^b^	220	58	218	272
hCG‐positive^b^	104 (47.3)	21 (36.2)	69 (31.7)**	88 (32.4)**
GS‐positive^b^	83 (37.7)	15 (25.9)	53 (24.3)*	66 (24.3)**
FHM‐positive^b^	77 (35.0)	12 (20.7)	49 (22.5)*	57 (21.0)**
LB/OP^b^	72 (32.7)	9 (15.5)*	45 (20.6)*	50 (18.4)**
Miscarriage^b^, ^e^	11 (13.3)	6 (40.0)	8 (15.1)	16 (24.2)

Abbreviations: FHM, fetal heart movement; GnRH, gonadotropin‐releasing hormone; GS, gestational sac; GV, germinal vesicle; hCG, human chorionic gonadotropin; ICSI, intracytoplasmic sperm injection; LB/OP, live birth or ongoing pregnancy; MI, metaphase I; MII, metaphase II; PB, polar body; PN, pronuclei; PPOS, progestin‐primed ovarian stimulation.

**p < 0.01, *p < 0.05, each protocol vs. PPOS protocol. ^§§^ *p* < 0.01, each protocol vs. long protocol. ^††^ *p* < 0.01, each protocol vs. short protocol.

^a^ Steel–Dwass test.

^b^ Holm test.

^c^ ICSI cycles using frozen‐thawed spermatozoa, spermatozoa retrieved by testicular sperm extraction, or oocytes that were artificially activated were excluded from the analysis.

^d^ 2PN2PB oocytes cultured until confirmation of blastocyst formation.

^e^ Rate of miscarriage = Patients with miscarriage (*n*)/GS‐positive patients (*n*) × 100.

When we examined the relationship between different COS protocols and clinical outcomes after ICSI, we excluded ICSI cycles using frozen‐thawed spermatozoa, spermatozoa retrieved by testicular sperm extraction, or oocytes that were artificially activated from the analysis. Again, female patients treated with the GnRH antagonist protocol (39.7 ± 4.8 years) were significantly older when OPU was performed than patients treated with the PPOS (36.1 ± 5.0 years), long (34.9 ± 4.1 years), and short (36.5 ± 4.0 years) protocols in this slightly smaller cohort (*p* < 0.01, Table 1). The rate of 2PN2PB oocytes from ICSI after the PPOS protocol (1666/2166, 76.9%) was significantly higher than after the long (254/367, 69.2%), short (1030/1418, 72.6%), or GnRH antagonist (1411/1943, 72.6%) protocols (*p* < 0.05, *p* < 0.01, and *p* < 0.01, respectively, Table 1). Additionally, the blastulation rate after ICSI with the PPOS protocol (997/1444, 69.0%) was significantly higher than after ICSI with the GnRH antagonist protocol (665/1062, 62.6%, *p* < 0.01, Table 1). Similarly, the rates of hCG‐positivity, GS‐positivity, and FHM‐positivity were significantly higher using the PPOS protocol (104/220, 47.3%; 83/220, 37.7%; and 77/220, 35.0%) prior to ICSI than with the short (69/218, 31.7%; 53/218, 24.3%; and 49/218, 22.5%) or GnRH antagonist (88/272, 32.4%; 66/272, 24.3%; and 57/272; 21.0%) protocols (*p* < 0.05, Table 1). The rates of LB/OP were significantly higher using the PPOS protocol (72/220, 32.7%) prior to ICSI than with the long (9/58, 15.5%, *p* < 0.05), short (45/218, 20.6%, *p* < 0.05), or GnRH antagonist (50/272, 18.4%, *p* < 0.01) protocols (Table 1). In contrast, the rates of miscarriage did not differ among the COS protocols (Table 1).

### 3.2 The relationship between meiotic spindle visibility and controlled ovarian stimulation protocols

First, we examined the relationship between spindle visibility and the hCG‐ICSI interval. We limited the analysis to patients with ICSI cycles conducted using the spindle‐view system. The rate of spindle visibility increased as the hCG‐ICSI interval increased (from 59.6% to 89.6%, Table 2). The hCG‐ICSI interval following the GnRH antagonist protocol (39.4 ± 2.1 h) was shorter than following the PPOS (40.1 ± 1.4 h, *p* < 0.01), long (40.5 ± 1.5 h, *p* < 0.01), and short (40.4 ± 1.5 h, *p* < 0.05) protocols, and the hCG‐ICSI interval following the PPOS protocol was shorter than that of the long and short protocols (*p* < 0.01, Table 2). When the relationship between COS protocol, spindle visibility, and hCG‐ICSI interval was examined, the rate of spindle visibility did not vary by hCG‐ICSI interval after the PPOS protocol (from 80.4% to 100.0%, Table 2). After COS with the long protocol, the rate of spindle visibility was significantly higher when the hCG‐ICSI interval was 42.0–42.9 h (100.0%) as compared with 37.0–39.9 h and ≥43 h (33.3%–83.2%) (Table 2). Using the short or antagonist protocols, the rate of spindle visibility increased as the hCG‐ICSI interval increased (short: from 20.0% to 91.4%, GnRH antagonist: from 60.4% to 88.6%, Table 2). When we compared COS protocols, the rates of spindle visibility when the hCG‐ICSI interval was 38.0–38.9 h were significantly higher using the short protocol (91.4%) as compared with any other protocol (71.0%–82.0%) (*p* < 0.05, Table 2). The rate of spindle visibility when the hCG‐ICSI interval was 42.0–42.9 h was significantly higher with the long protocol (100.0%) as compared with the PPOS (85.2%) or antagonist protocol (88.6%) (*p* < 0.01, Table 2).

**TABLE 2:** The relationship between the length of time from hCG administration to completion of ICSI and oocyte spindle visibility.

	Time from hCG administration to completion of ICSI (hours)^a^								
	<36	36.0–36.9	37.0–37.9	38.0–38.9	39.0–39.9	40.0–40.9	41.0–41.9	42.0–42.9	≥43
Total (hCG‐ICSI interval: 40.0 ± 1.6 h)									
Oocyte	114	201	313	898	1336	1175	979	521	200
Visible	68	146	240	742	1099	1032	862	467	173
%	59.6	72.6	76.7*	82.6**^,§^	82.3**^,§^	87.8**^,§§,††,#,¶¶^	88.0**^,§§,††,#,¶¶^	89.6**^,§§,††,##,¶¶^	86.5**^,§^
PPOS (hCG‐ICSI interval^b^: 40.1 ± 1.4 h)									
Oocyte	3	11	89	362	443	417	364	162	33
Visible	3	11	74	297	356	379	325	138	27
%	100.0	100.0	83.1	82.0	80.4	90.9	89.3	85.2	81.8
Long (hCG‐ICSI interval^b^: 40.5 ± 1.5 h)^x^									
Oocyte	0	0	3	69	119	57	65	63	20
Visible	0	0	1	49	99	52	56	63	15
%	–	–	33.3	71.0	83.2	91.2	86.2	100.0^†,##,¶¶,‡,x^	75.0
Short (hCG‐ICSI interval^b^: 40.4 ± 1.5 h)^x^									
Oocyte	5	9	39	162	324	365	252	138	77
Visible	1	7	26	148	273	316	224	126	69
%	20.0	77.8	66.7	91.4*^,††,x,y^	84.3	86.6*	88.9*^,†^	91.3*^,†^	89.6*
GnRH antagonist (hCG‐ICSI interval^b^: 39.4 ± 2.1 h)^x,y,z^									
Oocyte	106	181	182	305	450	336	298	158	70
Visible	64	128	139	248	371	285	257	140	62
%	60.4	70.7	76.4	81.3**^,z^	82.4**^,§^	84.8**^,§§^	86.2**^,§§^	88.6**^,§§,y^	88.6**

Abbreviations: GnRH, gonadotropin‐releasing hormone; hCG‐ICSI interval, interval from human chorionic gonadotropin administration to the completion of intracytoplasmic sperm injection; PPOS, progestin‐primed ovarian stimulation.

**p < 0.01, *p < 0.05, each group vs. <36 h. ^§§^ *p* < 0.01, ^§^ *p* < 0.05, each group vs. 36‐36.9 h. ^††^ *p* < 0.01, ^†^ *p* < 0.05, each group vs. 37–37.9 h. ^##^ *p* < 0.01, ^#^ *p* < 0.05, each group vs. 38–38.9 h. ^¶¶^ *p* < 0.01, each group vs. 39–39.9 h. ^‡^ *p* < 0.05, each group vs. ≥43 h. ^x^ *p* < 0.01, each protocol vs. PPOS protocol. ^y^ *p* < 0.01 each protocol vs. long protocol. ^z^ *p* < 0.05 each protocol vs. short protocol.

^a^ Holm test.

^b^ Steel‐Dwass.

Next, we investigated the impact of meiotic spindle visibility in the oocyte on clinical outcomes after ICSI with each COS protocol and the potential relationship between COS protocol and meiotic spindle visibility. Again, we limited the analysis to patients with ICSI cycles conducted using the spindle‐view system. Oocytes with visible spindle were obtained from women who were significantly younger than the women from whom oocytes with a nonvisible spindle were obtained (35.8 ± 4.8 years vs. 36.8 ± 5.1 years, *p* < 0.001, Table 3). The meiotic spindle was visible in the oocyte significantly more often after the PPOS (1610/1884, 85.5%) or short (1190/1371, 86.8%) protocols than after the GnRH antagonist protocol (1694/2086, 81.2%) (*p* < 0.01 and *p* < 0.05, respectively, Table 3). Progression to the 2PN2PB stage was significantly higher in oocytes with visible spindles (PPOS, 1157/1479, 78.2%; long, 209/290, 72.1%; short, 840/1096, 76.6%; and GnRH antagonist; 1125/1459, 77.1%) than in oocytes with nonvisible spindles (PPOS, 149/241, 61.8%; long, 20/46, 43.5%; short, 83/156, 53.2%; and GnRH antagonist; 170/319, 53.3%) after each of the COS protocols (*p* < 0.01), and there were no significant differences in the 2PN2PB rates between COS protocols in oocytes with a visible spindles (Table 3). Using the GnRH antagonist protocol, the blastulation rate in embryos derived from oocytes with visible spindles (568/865, 65.7%) was significantly higher than that of embryos derived from oocytes with nonvisible spindles (47/112, 42.0%, *p* < 0.001, Table 3). For all other protocols, blastulation was not significantly associated with spindle visibility. When we compared blastulation rates between the COS protocols, there were no significant differences observed for oocytes with visible spindles, but the rates of blastulation from oocytes with nonvisible spindles using the PPOS (84/127, 66.1%) or short (46/72, 63.9%) protocols were significantly higher than blastulation rates using the GnRH antagonist protocol (47/112, 42.0%) (*p* < 0.01 and *p* < 0.05, respectively, Table 3). There were no significant differences in the rates of GS‐positivity, LB/OP, or miscarriage between the visible and nonvisible spindle groups (Table 3). When we compared each COS protocol, the rate of LB/OP from oocytes with a visible spindle after PPOS (48/155, 31.0%) was significantly higher than that from oocytes with a visible spindle after the GnRH antagonist protocol (43/230, 18.7%, *p* < 0.05, Table 3).

**TABLE 3:** The relationship between the visibility of meiotic spindle in the oocyte and controlled ovarian stimulation protocol.

Outcome/COS protocol	Total	Visible spindle	Nonvisible spindle	*p* value
Female age at OPU, mean years ± SD		35.8 ± 4.8	36.8 ± 5.1	<0.001^a^
	*n* (%)	*n* (%)	*n* (%)	
MII oocytes^b^, ^c^				
Total	5737	4829 (84.2)	908 (15.8)	–
PPOS	1884	1610 (85.5)	274 (14.5)	–
Long	396	335 (84.6)	61 (15.4)	–
Short	1371	1190 (86.8)	181 (13.2)	–
GnRH antagonist	2086	1694 (81.2)**^,†^	392 (18.8)**^,†^	–
2PN2PB oocytes/MII oocytes with ICSI^d^, ^e^				
Total	3753/5086 (73.8)	3331/4324 (77.0)	422/762 (55.4)	<0.001^f^
PPOS	1306/1720 (75.9)	1157/1479 (78.2)	149/241 (61.8)	<0.001^f^
Long	229/336 (68.2)	209/290 (72.1)	20/46 (43.5)	<0.001^f^
Short	923/1252 (73.7)	840/1096 (76.6)	83/156 (53.2)	<0.001^f^
GnRH antagonist	1295/1778 (72.8)	1125/1459 (77.1)	170/319 (53.3)	<0.001^f^
Embryo development				
Blastocysts/2PN2PB oocytes cultured until confirmation of blastocyst formation^e^				
Total	2020/3079 (65.6)	1835/2751 (66.7)	185/328 (56.4)	<0.001^f^
PPOS	764/1130 (67.6)	680/1003 (67.8)	84/127 (66.1)	0.763^f^
Long	133/197 (67.5)	125/180 (69.4)	8/17 (47.1)	0.101^f^
Short	508/775 (65.5)	462/703 (65.7)	46/72 (63.9)	0.795^f^
GnRH antagonist	615/977 (62.9)	568/865 (65.7)	47/112 (42.0)**^,†^	<0.001^f^
Pregnancy, live birth, and miscarriage				
Clinical Pregnancy (gestational sac‐positive)/Blastocyst transfer cycles^e^				
Total	181/672 (26.9)	163/612 (26.6)	18/60 (30.0)	0.546^f^
PPOS	60/173 (34.7)	55/155 (35.5)	5/18 (27.8)	0.608^f^
Long	15/55 (27.3)	14/51 (27.5)	1/4 (25.0)	1.000^f^
Short	47/195 (24.1)	41/176 (23.3)	6/19 (31.6)	0.408^f^
GnRH antagonist	59/249 (23.7)	53/230 (23.0)	6/19 (31.6)	0.405^f^
Live birth or ongoing pregnancy/Blastocyst transfer cycles^e^				
Total	151/672 (22.5)	136/612 (22.2)	15/60 (25.0)	0.628^f^
PPOS	53/173 (30.6)	48/155 (31.0)	5/18 (27.8)	1.000^f^
Long	9/55 (16.4)	8/51 (15.7)	1/4 (25.0)	0.522^f^
Short	42/195 (21.5)	37/176 (21.0)	5/19 (26.3)	0.565^f^
GnRH antagonist	47/249 (18.9)	43/230 (18.7)*	4/19 (21.1)	0.764^f^
Miscarriage^g^				
Total	30/181 (16.6)	27/163 (16.6)	3/18 (16.7)	1.000^f^
PPOS	7/60 (11.7)	7/55 (12.7)	0/5 (0.0)	1.000^f^
Long	6/15 (40.0)	6/14 (42.9)	0/1 (0.0)	1.000^f^
Short	5/47 (10.6)	4/41 (9.8)	1/6 (16.7)	0.511^f^
GnRH antagonist	12/59 (20.3)	10/53 (18.9)	2/6 (33.3)	0.591^f^

Abbreviations: COS, controlled ovarian stimulation; GnRH, gonadotropin‐releasing hormone; ICSI, intracytoplasmic sperm injection; MII, metaphase II; PB, polar body; PN, pronuclei; PPOS, progestin‐primed ovarian stimulation.

**p < 0.01, *p < 0.05 each protocol vs. PPOS protocol. ^†^ *p* < 0.05 each protocol vs. short protocol.

^a^ Mann–Whitney U test.

^b^ Holm test.

^c^ Oocyte with first polar body regardless of visibility or any position of spindle.

^d^ ICSI cycles using frozen‐thawed spermatozoa, spermatozoa retrieved by testicular sperm extraction, or oocytes that were artificially activated were excluded from the analysis.

^e^ Comparison between each COS protocol in visible or nonvisible of spindle groups by Holm test.

^f^ Fisher's exact test.

^g^ Rate of miscarriage = Patients with miscarriage (*n*)/GS‐positive patients (*n*) × 100.

To confirm that the factors identified above were correlated with spindle visibility, we analyzed female age at the time of OPU, COS protocol, and the hCG‐ICSI interval in a univariant logistic analysis. Both the PPOS and short protocols had higher odds of a visible spindle (PPOS, odds ratio [OR], 1.36; 95% CI, 1.15–1.61; *p* = 0.0004; short, OR, 1.52; 95% CI, 1.26–1.84; *p* < 0.0001) as compared with the GnRH antagonist protocol, which was used as the reference (Table 4). Female age at the time of OPU (OR, 0.96; 95% CI, 0.946–0.975; *p* < 0.0001) and the hCG‐ICSI interval (OR, 1.23; 95% CI, 1.18–1.28; *p* < 0.0001) were also associated with spindle visibility (Table 4). In a multiple logistic regression analysis, the short protocol had higher odds of a visible spindle (OR, 1.24; 95% CI, 1.02–1.51; *p* < 0.0330) as compared with the GnRH antagonist protocol, which was used as the reference (Table 4). The hCG‐ICSI interval was also associated with spindle visibility (OR, 1.20; 95% CI, 1.14–1.25; *p* < 0.0001, Table 4).

**TABLE 4:** Logistic analysis of the relationship between spindle visibility and controlled ovarian stimulation protocol^a^.

Variable	*β*	SE *β*	OR	95% CI	*p*‐value
Univariate logistic analysis					
Female (oocyte provider) age^b^	−0.040	0.008	0.960	0.946–0.975	<0.0001
PPOS^c^	0.307	0.086	1.360	1.150–1.610	<0.001
Long^c^	0.240	0.150	1.270	0.947–1.710	0.110
Short^c^	0.420	0.098	1.520	1.260–1.840	<0.0001
hCG‐ICSI interval^b^	0.203	0.020	1.230	1.180–1.280	<0.0001
Multivariate logistic analysis					
Female (oocyte provider) age^b^	−0.009	0.008	0.991	0.975–1.010	0.303
PPOS^c^	0.149	0.090	1.160	0.973–1.380	0.098
Long^c^	0.005	0.154	1.000	0.743–1.360	0.976
Short^c^	0.216	0.101	1.240	1.020–1.510	0.033
hCG‐ICSI interval^b^	0.181	0.023	1.200	1.140–1.250	<0.0001

*Note*: Multiple logistic regression analysis.

Abbreviations: *β*, regression coefficient; CI, confidence interval; GnRH, gonadotropin‐releasing hormone; hCG, human chorionic gonadotropin; hCG‐ICSI interval, interval from hCG administration to the completion of ICSI; ICSI, intracytoplasmic sperm injection; OR, odds ratio; PPOS, progestin‐primed ovarian stimulation; SE *β*, standard error of the regression coefficient.

^a^ Reference: nonvisible spindle.

^b^ Female age and the hCG‐ICSI interval were considered as continuous variables.

^c^ Reference: GnRH antagonist protocol.

### 3.3 The relationship between controlled ovarian stimulation protocol and the position of the meiotic spindle relative to the polar body

Next, we investigated the relationship between the COS protocol and the position of the meiotic spindle relative to the PB in oocytes with a visible spindle. The spindle was positioned at 0° in the MII oocytes at a significantly higher rate after the PPOS protocol (853/1884, 45.3%) than after the short (543/1371, 39.6%) or GnRH antagonist (792/2086, 38.0%) protocols (*p* < 0.01, Table 5). At all other spindle positions relative to the PB (0° < *θ* ≤ 30°, 30° < *θ* ≤ 60°, 60° < *θ* ≤ 90°, and 90° < *θ* ≤ 180°), there were no significant differences in positioning in MII oocytes among the different COS protocols (Table 5). Additionally, the PPOS and short protocols had significantly fewer meiotic spindles that were located between the PB and the oolemma or that were nonvisible as compared with the GnRH antagonist protocol (spindle between the PB and oolemma: PPOS, 11/1884, 0.6% and short, 7/1371, 0.5% vs. antagonist, 33/2086, 1.6%, *p* < 0.05; nonvisible spindle: PPOS, 274/1884, 14.5% and short, 181/1371, 13.2% vs. antagonist 392/2086, 18.8%, *p* < 0.05; Table 5). Then, we investigated the relationship between the position of the meiotic spindle relative to the PB and female age at OPU. The spindle was positioned at 0° in the MII oocytes from women younger than 30 years at a significantly higher rate than in MII oocytes from women from ≥45 years of age (<30 years, 250/582, 43.0%, and 30–34 years, 717/1618, 44.3% vs. >45 years, 56/179, 31.3%, *p* < 0.05 and *p* < 0.01, respectively, Table 6). Additionally, the spindle was positioned at 0° in the MII oocytes at a significantly higher rate in the oocytes from 30‐to‐34‐year‐old women (717/1618, 44.3%) than from women who were older than 40 years of age (474/1265, *p* < 0.01, Table 6). Conversely, there were significantly fewer MII oocytes with nonvisible spindles from women <45 years of age than from women older than ≧45 years (<30 years, 75/582, 12.9%; 30–34 years, 222/1618, 13.7%, 35–39 years, 334/2093, 16.0%; and 40–44 years, 217/1265, 17.2% vs. ≥45 years, 60/179, 33.5%, *p* < 0.01, Table 6).

**TABLE 5:** The relationship between the controlled ovarian stimulation protocols and position (*θ*) of the meiotic spindle relative to the polar body.

Spindle position	Controlled ovarian stimulation protocol			
	PPOS	Long	Short	GnRH antagonist
	*n* (%)	*n* (%)	*n* (%)	*n* (%)
MII oocytes^a^	1884	396	1371	2086
*θ* = 0°	853 (45.3)	166 (41.9)	543 (39.6)**	792 (38.0)**
0° < *θ* ≤ 30°	375 (19.9)	81 (20.5)	311 (22.7)	450 (21.6)
30° < *θ* ≤ 60°	235 (12.5)	49 (12.4)	197 (14.4)	252 (12.1)
60° < *θ* ≤ 90°	89 (4.7)	23 (5.8)	88 (6.4)	125 (6.0)
90° < *θ* ≤ 180°	47 (2.5)	10 (2.5)	44 (3.2)	42 (2.0)
PB/oolemma^b^	11 (0.6)	6 (1.5)	7 (0.5)	33 (1.6)*^,†^
Nonvisible spindle	274 (14.5)	61 (15.4)	181 (13.2)	392 (18.8)*^,†^

*Note*: Holm test used to determine significance.

Abbreviations: GnRH, gonadotropin‐releasing hormone; MII, metaphase II; PB, polar body; PPOS, progestin‐primed ovarian stimulation.

**p < 0.01, *p < 0.05 each protocol vs. PPOS protocol. ^†^ *p* < 0.05 each protocol vs. short protocol.

^a^ Oocyte with first polar body regardless of visibility or any position of spindle.

^b^ Spindle bridge between the first polar body and the oolemma.

**TABLE 6:** The relationship between the female age at OPU and position (*θ*) of the meiotic spindle relative to the polar body.

	Female age (years)				
	<30	30–34	35–39	40–44	≥45
	*n* (%)	*n* (%)	*n* (%)	*n* (%)	*n* (%)
MII oocyte^a^	582	1618	2093	1265	179
*θ* = 0°	250 (43.0)	717 (44.3)	857 (40.9)	474 (37.5)^§§^	56 (31.3)*^,§§^
0° < *θ* ≤ 30°	137 (23.5)	360 (22.2)	431 (20.6)	259 (20.5)	30 (16.8)
30° < *θ* ≤ 60°	71 (12.2)	201 (12.4)	259 (12.4)	180 (14.2)	22 (12.3)
60° < *θ* ≤ 90°	24 (4.1)	77 (4.8)	139 (6.6)	80 (6.3)	5 (2.8)
90° < *θ* ≤ 180°	13 (2.2)	31 (1.9)	59 (2.8)	36 (2.8)	4 (2.2)
PB/other	12 (2.1)	10 (0.6)	14 (0.7)	19 (1.5)	2 (1.1)
Nonvisible spindle	75 (12.9)	222 (13.7)	334 (16.0)	217 (17.2)	60 (33.5)**^,§§,††,##^

*Note*: Holm test used to determine significance.

Abbreviations: MII, metaphase II; OPU, ovum pick up; PB, polar body.

**p <0.01, *p < 0.05, each group vs. <30 years. ^§§^ *p* < 0.01, each group vs. 30–34 years. ^††^ *p* < 0.01, each group vs. 35–39 years. ^##^ *p* < 0.01, each group vs. 40–44 years.

^a^ Oocyte with first polar body regardless of visibility or any position of spindle.

Univariate multinominal logistic regression analysis showed that female age, COS protocol, and the hCG‐ICSI interval impacted the position of the meiotic spindle (Table 7). In the logistic regression analysis, the woman's age at the time of OPU was associated with spindle position, and the odds of a spindle located at 30° < *θ* ≤ 60°, at 60° < *θ* ≤ 90°, or at 90° < *θ* ≤ 180° were increased relative to the *θ* = 0° in older women (Table 8; 30° < *θ* ≤ 60°, OR, 1.020, 95% CI, 1.000–1.040; *p* = 0.042; 60° < *θ* ≤ 90°, OR, 1.030, 95% CI, 1.010–1.060; *p* = 0.013; 90° < *θ* ≤ 180°, OR, 1.060, 95% CI, 1.020–1.100, *p* = 0.005). The odds of spindle positioning 0° < *θ* ≤30° or 60° < *θ* ≤ 90° were lower after PPOS as compared with the GnRH antagonist protocol (Table 8; 0° < *θ* ≤ 30°, OR, 0.76, 95% CI, 0.641–0.902; *p* = 0.002; 60° < *θ* ≤ 90°, OR, 0.690, 95% CI, 0.514–0.926; *p* = 0.013). The hCG‐ICSI interval was associated with spindle position (Table 8; 30° < *θ* ≤ 60°, OR, 1.060, 95% CI, 1.000–1.012; *p* = 0.046). Spindle positioning between the PB and the oolemma or a nonvisible spindle associated with the PPOS and short protocols and the hCG‐ICSI interval (Table 8).

**TABLE 7:** Univariate logistic regression analysis of factors associated the position (*θ*) of the meiotic spindle relative to the polar body.

Variable	Spindle position^a^	*β*	SE*β*	Wald	OR	95% CI	*p*‐value
Female (oocyte provider) age^b^	0° < *θ* ≤ 30°	0.0005	0.007	0.065	1.000	0.986–1.010	0.948
	30° < *θ* ≤ 60°	0.014	0.009	1.645	1.010	0.997–1.030	0.100
	60° < *θ* ≤ 90°	0.031	0.012	2.511	1.030	1.010–1.06	0.012
	90° < *θ* ≤ 180°	0.042	0.018	2.377	1.040	1.010–1.080	0.017
	PB/oolemma^d^	0.013	0.008	0.486	1.010	0.960–1.070	0.627
	Nonvisible	0.046	0.008	5.714	1.050	1.030–1.060	<0.0001
PPOS^c^	0° < *θ* ≤ 30°	−0.257	0.086	−2.998	0.774	0.654–0.915	0.003
	30° < *θ* ≤ 60°	−0.144	0.103	−1.395	0.866	0.707–1.060	0.163
	60° < *θ* ≤ 90°	−0.414	0.147	−2.811	0.661	0.495–0.882	0.005
	90° < *θ* ≤ 180°	0.038	0.218	0.176	1.040	0.678–1.590	0.861
	PB/oolemma^d^	−1.173	0.352	−3.335	0.309	0.155–0.617	<0.0001
	Nonvisible	−0.432	0.093	−4.652	0.649	0.541–0.779	<0.0001
Long^c^	0° < *θ* ≤ 30°	−0.152	0.148	−1.030	0.859	0.643–1.150	0.303
	30° < *θ* ≤ 60°	−0.075	0.178	−0.422	0.928	0.655–1.310	0.673
	60° < *θ* ≤ 90°	−0.130	0.242	−0.537	0.878	0.546–1.410	0.591
	90° < *θ* ≤ 180°	0.128	0.362	0.352	1.140	0.559–2.310	0.725
	PB/oolemma^d^	−0.142	0.452	−0.315	0.867	0.358–2.100	0.753
	Nonvisible	−0.298	0.162	−1.839	0.742	0.540–1.020	0.066
Short^c^	0° < *θ* ≤ 30°	0.008	0.092	0.086	1.010	0.841–1.210	0.931
	30° < *θ* ≤ 60°	0.131	0.110	1.191	1.140	0.919–1.420	0.234
	60° < *θ* ≤ 90°	0.026	0.150	0.177	1.030	0.765–1.380	0.860
	90° < *θ* ≤ 180°	0.424	0.223	1.903	1.530	0.987–2.360	0.057
	PB/oolemma^d^	−1.173	0.420	−2.794	0.309	0.136–0.705	0.005
	Nonvisible	−0.395	0.106	−3.739	0.673	0.547–0.829	<0.001
hCG‐ICSI interval^b^	0° < *θ* ≤ 30°	0.034	0.021	1.632	1.030	0.993–1.080	0.103
	30° < *θ* ≤ 60°	0.034	0.025	1.370	1.030	0.985–1.090	0.171
	60° < *θ* ≤ 90°	0.019	0.035	0.535	1.020	0.951–1.090	0.593
	90° < *θ* ≤ 180°	0.032	0.051	0.623	1.030	0.934–1.140	0.533
	PB/oolemma^d^	−0.256	0.071	−3.591	0.774	0.673–0.890	<0.0001
	Nonvisible	−0.192	0.022	−8.640	0.826	0.790–0.862	<0.0001

*Note*: Univariate multinominal logistic regression analysis.

Abbreviations: *β*, regression coefficient; CI, confidence interval; GnRH, gonadotropin‐releasing hormone; hCG, human chorionic gonadotropin; hCG‐ICSI interval, from hCG administration to the completion of ICSI; ICSI, intracytoplasmic sperm injection; OR, odds ratio; PPOS, progestin‐primed ovarian stimulation; SE *β*, standard error of the regression coefficient; *θ*, relative position of the meiotic spindle to the polar body.

^a^ Reference: *θ* = 0°.

^b^ Female age and the hCG‐ICSI interval were considered as a continuous variable.

^c^ Reference GnRH antagonist protocol.

^d^ Spindle bridge between the first polar body and the oolemma.

**TABLE 8:** Multinominal logistic regression analysis of the relationship between the position (*θ*) of the meiotic spindle relative to the polar body and COS protocol.

Spindle position	Variable	*β*	SE*β*	Wald	OR	95% CI	*p*‐value
0° < *θ* ≤ 30°^a^	Female (oocyte provider) age^b^	0.001	0.008	0.172	1.000	0.986–1.020	0.863
	PPOS^c^	−0.274	0.087	−3.142	0.760	0.641–0.902	0.002
	Long^c^	−0.187	0.150	−1.252	0.829	0.618–1.110	0.211
	Short^c^	−0.024	0.094	−0.258	0.976	0.812–1.170	0.797
	hCG‐ICSI interval^b^	0.041	0.023	1.779	1.040	0.996–1.090	0.075
30° < *θ* ≤ 60°^a^	Female (oocyte provider) age^b^	0.020	0.010	2.032	1.020	1.000–1.040	0.042
	PPOS^c^	−0.129	0.105	−1.228	0.879	0.715–1.080	0.219
	Long^c^	−0.078	0.180	−0.434	0.925	0.649–1.320	0.664
	Short^c^	0.105	0.112	0.932	1.110	0.891–1.380	0.352
	hCG‐ICSI interval^b^	0.055	0.028	1.992	1.060	1.000–1.120	0.046
60° < *θ* ≤ 90°^a^	Female (oocyte provider) age^b^	0.034	0.014	2.484	1.030	1.010–1.060	0.013
	PPOS^c^	−0.371	0.150	−2.472	0.690	0.514–0.926	0.013
	Long^c^	−0.102	0.246	−0.416	0.903	0.557–1.460	0.677
	Short^c^	0.010	0.153	0.064	1.010	0.748–1.360	0.949
	hCG‐ICSI interval^b^	0.062	0.038	1.605	1.060	0.986–1.150	0.108
90° < *θ* ≤ 180°^a^	Female (oocyte provider) age^b^	0.057	0.020	2.787	1.060	1.020–1.100	0.005
	PPOS^c^	0.123	0.223	0.551	1.130	0.731–1.750	0.582
	Long^c^	0.202	0.368	0.550	1.220	0.595–2.520	0.582
	Short^c^	0.421	0.229	1.842	1.520	0.973–2.380	0.065
	hCG‐ICSI interval^b^	0.076	0.057	1.344	1.080	0.966–1.210	0.179
PB/oolemma^a^, ^d^	Female (oocyte provider) age^b^	−0.047	0.030	−1.566	0.954	0.900–1.010	0.117
	PPOS^c^	−1.103	0.363	−3.039	0.332	0.163–0.676	0.002
	Long^c^	0.020	0.464	0.043	1.020	0.411–2.540	0.966
	Short^c^	−0.973	0.429	−2.269	0.378	0.163–0.876	0.023
	hCG‐ICSI interval^b^	−0.257	0.081	−3.167	0.773	0.659–0.907	0.002
Nonvisible^a^	Female (oocyte provider) age^b^	0.015	0.009	1.675	1.020	0.997–1.030	0.094
	PPOS^c^	−0.271	0.097	−2.803	0.762	0.631–0.922	0.005
	Long^c^	−0.067	0.166	−0.401	0.936	0.676–1.290	0.688
	Short^c^	−0.204	0.109	−1.867	0.815	0.658–1.010	0.062
	hCG‐ICSI interval^b^	−0.160	0.025	−6.313	0.852	0.811–0.896	<0.0001

*Note*: Multinominal logistic regression analysis.

Abbreviations: *β*, regression coefficient; CI, confidence interval; COS, controlled ovarian stimulation; GnRH, gonadotropin‐releasing hormone; hCG, human chorionic gonadotropin; hCG‐ICSI interval, from hCG administration to the completion of ICSI; ICSI, intracytoplasmic sperm injection; OR, odds ratio; PPOS, progestin‐primed ovarian stimulation; SE *β*, standard error of the regression coefficient; *θ*, relative position of the meiotic spindle to the polar body.

^a^ Reference: *θ* = 0°.

^b^ Female age and the hCG‐ICSI interval were considered as a continuous variable.

^c^ Reference GnRH antagonist protocol.

^d^ Spindle bridge between the first polar body and the oolemma.

### 3.4 The relationship between the position of the meiotic spindle relative to the polar body and clinical outcomes

To investigate the relationship between the position of the meiotic spindle relative to the PB and clinical outcomes, oocytes with spindle position at 0° ≤ *θ* ≤ 180° were analyzed. Oocytes with a PB/oolemma or with a nonvisible spindle were excluded; these oocytes were likely in anaphase I/telophase I or interkinesis, respectively.^23^ The women who provided oocytes with spindle positions of 90° < *θ* ≤ 180° were significantly older (37.0 ± 4.7 years) than the women who provided oocytes with spindle positions of *θ* = 0° (35.5 ± 4.7 years) or 0° < *θ* ≤ 30° (35.7 ± 4.8 years) (*p* < 0.01 and *p* < 0.05, respectively, Table 9). Additionally, the women who provided oocytes with spindle positions of 60° < *θ* ≤ 90° (36.4 ± 4.5 years) were significantly older than the women who provided oocytes with spindle position of *θ* = 0° (35.5 ± 4.7 years) (*p* < 0.05, Table 9). The rates of 2PN2PB, 1PN, multi‐PN, and no‐PN oocyte formation after ICSI were not significantly different among oocytes with different spindle positions (Table 9). However, the rate of unfertilized oocytes was significantly higher in oocytes with spindle positioning of *θ* = 0° (190/2097, 9.1%) as compared with oocytes with a spindle position of 0° < *θ* ≤ 30° (63/1087, 5.8%, *p* < 0.05, Table 9). The rate of broken oocytes was significantly higher in oocytes with a spindle position of 90° < *θ* ≤ 180° (17/127, 13.4%) as compared with oocytes with spindle position of *θ* = 0° (103/2097, 4.9%, *p* < 0.01) and 0° < *θ* ≤ 30° (57/1087, 5.2%, *p* < 0.05, Table 9). The rates of blastulation, euploidy, hCG‐positivity, GS‐positivity, FHM‐positivity, LB/OP, and miscarriage did not differ based on spindle position (Table 9).

**TABLE 9:** The relationship between the position (*θ*) of the meiotic spindle relative to the polar body and clinical outcomes after ICSI.

Outcome	*θ* = 0°	0° < *θ* ≤ 30°	30° < *θ* ≤ 60°	60° < *θ* ≤ 90°	90° < *θ* ≤ 180°
Female age, mean years ± SD^a^	35.5 ± 4.7	35.7 ± 4.8	36.1 ± 4.8	36.4 ± 4.5*	37.0 ± 4.7**^,§^
	*n* (%)	*n* (%)	*n* (%)	*n* (%)	*n* (%)
ICSI^b^					
MII oocytes^c^	2097	1087	672	291	127
2PN2PB oocytes	1613 (76.9)	874 (80.4)	511 (76.0)	216 (74.2)	93 (73.2)
1PN oocytes	42 (2.0)	21 (1.9)	19 (2.8)	14 (4.8)	4 (3.1)
Multi‐PN oocytes	62 (3.0)	29 (2.7)	26 (3.9)	12 (4.1)	4 (3.1)
No‐PN oocytes	87 (4.1)	43 (4.0)	31 (4.6)	12 (4.1)	3 (2.4)
Unfertilized oocytes	190 (9.1)	63 (5.8)*	43 (6.4)	18 (6.2)	6 (4.7)
Broken oocytes	103 (4.9)	57 (5.2)	42 (6.3)	19 (6.5)	17 (13.4)**^,§^
Embryo development					
Cultured 2PN2PB^d^	1362	715	413	167	73
Blastocysts	906 (66.5)	486 (68.0)	266 (64.4)	120 (71.9)	46 (63.0)
Ploidy status					
Analyzed blastocysts	62	47	14	7	2
Euploid	27 (43.5)	15 (31.9)	9 (64.3)	1 (14.3)	0 (0.0)
Aneuploid	35 (56.5)	32 (68.1)	5 (35.7)	6 (85.7)	2 (100.0)
Pregnancy, live birth, and miscarriage					
Blastocyst transfer cycles	307	153	100	41	13
hCG‐positive	116 (37.8)	53 (34.6)	31 (31.0)	14 (34.1)	4 (30.8)
GS‐positive	83 (27.0)	46 (30.1)	24 (24.0)	8 (19.5)	4 (30.8)
FHM‐positive	77 (25.1)	43 (28.1)	22 (22.0)	8 (19.5)	2 (15.4)
LB/OP	71 (23.1)	38 (24.8)	21 (21.0)	7 (17.1)	1 (7.7)
Miscarriage^e^	12 (14.5)	8 (17.4)	3 (12.5)	1 (12.5)	3 (75.0)

*Note*: Holm test used to determine significance.

Abbreviations: FHM, fetal heart movement; GS, gestational sac; hCG, human chorionic gonadotropin; ICSI, intracytoplasmic sperm injection; LB/OP, live birth or ongoing pregnancy; MII, metaphase II; PB, polar body; PN, pronuclei.

**p < 0.01, *p < 0.05, each group vs. *θ* = 0°. ^§^ *p* < 0.05, each group vs. 0° < *θ* ≤ 30°.

^a^ Steel–Dwass test.

^b^ ICSI cycles using frozen–thawed spermatozoa, spermatozoa retrieved by testicular sperm extraction, or oocytes that were artificially activated were excluded from the analysis.

^c^ Oocyte with spindle within ooplasm.

^d^ 2PN2PB oocytes cultured until confirmation of blastocyst formation.

^e^ Rate of miscarriage = Patients with miscarriage (*n*)/GS‐positive patients (*n*) × 100.

Finally, we analyzed the relationship between spindle position and clinical outcomes after ICSI separately for each COS protocol. Using the PPOS protocol, women who provided oocytes with spindle positions of 60° < *θ* ≤ 90° (36.0 ± 3.8 years) or 90° < *θ* ≤ 180° (36.6 ± 4.9 years) were significantly older than the women who provided oocytes with spindle positions of *θ* = 0° (34.4 ± 4.6 years, *p* < 0.05) or 0° < *θ* ≤ 30° (34.0 ± 4.7 years, *p* < 0.01) (Table 10). The women who provided oocytes with spindle positions of 30° < *θ* ≤ 60° were significantly older (35.2 ± 4.7 years) than the women who provided oocytes with spindle positions of 0° < *θ* ≤ 30° (34.0 ± 4.7 years) (*p* < 0.05, Table 10). Using long, short, or GnRH antagonist protocols, the spindle position did not differ with age at the time of OPU (Tables 11, 12, 13).

**TABLE 10:** The relationship between the PPOS protocol, position (*θ*) of the meiotic spindle relative to the polar body, and outcomes.

Outcome	*θ* = 0°	0° < *θ* ≤ 30°	30° < *θ* ≤ 60°	60° < *θ* ≤ 90°	90° < *θ* ≤ 180°
Female age, mean years ± SD^a^	34.4 ± 4.6	34.0 ± 4.7	35.2 ± 4.7^§^	36.0 ± 3.8*^,§§^	36.6 ± 4.9*^,§§^
	*n* (%)	*n* (%)	*n* (%)	*n* (%)	*n* (%)
ICSI^b^					
MII oocytes^c^	770	350	222	81	46
2PN2PB oocytes	606 (78.7)	289 (82.6)	168 (75.7)	57 (70.4)	32 (69.6)
1PN oocytes	18 (2.3)	9 (2.6)	6 (2.7)	6 (7.4)	4 (8.7)
Multi‐PN oocytes	19 (2.5)	7 (2.0)	6 (2.7)	3 (3.7)	1 (2.2)
No‐PN oocytes	35 (4.5)	14 (4.0)	9 (4.1)	3 (3.7)	0 (0.0)
Unfertilized oocytes	59 (7.7)	19 (5.4)	18 (8.1)	5 (6.2)	3 (6.5)
Broken oocytes	33 (4.3)	12 (3.4)	15 (6.8)	7 (8.6)	6 (13.0)
Embryo development					
Cultured 2PN2PB^d^	543	244	138	47	27
Blastocysts	367 (67.6)	172 (70.5)	90 (65.2)	34 (72.3)	14 (51.9)
Pregnancy, live birth, and miscarriage					
Blastocyst transfer cycles	79	42	25	7	1
hCG‐positive	40 (50.6)	16 (38.1)	10 (40.0)	3 (42.9)	0 (0.0)
GS‐positive	31 (39.2)	13 (31.0)	8 (32.0)	3 (42.9)	–
FHM‐positive	29 (36.7)	13 (31.0)	7 (28.0)	3 (42.9)	–
LB/OP	28 (35.4)	11 (26.2)	6 (24.0)	3 (42.9)	–
Miscarriage^e^	3 (9.7)	2 (15.4)	2 (25.0)	0 (0.0)	–

*Note*: Holm test used to determine significance.

Abbreviations: FHM, fetal heart movement; GS, gestational sac; hCG, human chorionic gonadotropin; ICSI, intracytoplasmic sperm injection; LB/OP, live birth or ongoing pregnancy; MII, metaphase II; PB, polar body; PN, pronuclei; PPOS, progestin‐primed ovarian stimulation.

*p < 0.05, each group vs. *θ* = 0°. ^§§^ *p* < 0.01, ^§^ *p* < 0.05, each group vs. 0° < *θ* ≤ 30°.

^a^ Steel–Dwass test.

^b^ ICSI cycles using frozen‐thawed spermatozoa, spermatozoa retrieved by testicular sperm extraction, or oocytes that were artificially activated were excluded from the analysis.

^c^ Oocyte with spindle within ooplasm.

^d^ 2PN2PB oocytes cultured until confirmation of blastocyst formation.

^e^ Rate of miscarriage = Patients with miscarriage (*n*)/GS‐positive patients (*n*) × 100.

**TABLE 11:** The relationship between the long protocol, position (*θ*) of the meiotic spindle relative to the polar body, and outcomes.

Outcome	*θ* = 0°	0° < *θ* ≤ 30°	30° < *θ* ≤ 60°	60° < *θ* ≤ 90°	90° < *θ* ≤ 180°
Female age, mean years ± SD^a^	34.7 ± 3.8	35.4 ± 3.7	33.5 ± 3.9	33.7 ± 4.4	36.0 ± 4.1
	*n* (%)	*n* (%)	*n* (%)	*n* (%)	*n* (%)
ICSI^b^					
MII oocytes^c^	154	61	42	20	7
2PN2PB oocytes	106 (68.8)	47 (77.0)	33 (78.6)	16 (80.0)	5 (71.4)
1PN oocytes	0 (0.0)	0 (0.0)	0 (0.0)	0 (0.0)	0 (0.0)
Multi‐PN oocytes	4 (2.6)	3 (4.9)	1 (2.4)	2 (10.0)	0 (0.0)
No‐PN oocytes	10 (6.5)	3 (4.9)	3 (7.1)	1 (5.0)	0 (0.0)
Unfertilized oocytes	26 (16.9)	4 (6.6)	4 (9.5)	1 (5.0)	1 (14.3)
Broken oocytes	8 (5.2)	4 (6.6)	1 (2.4)	0 (0.0)	1 (14.3)
Embryo development					
Cultured 2PN2PB^d^	90	42	27	15	5
Blastocysts	65 (72.2)	30 (71.4)	14 (51.9)	10 (66.7)	5 (100.0)
Pregnancy, live birth, and miscarriage					
Blastocyst transfer cycles	31	10	7	3	3
hCG‐positive	12 (38.7)	6 (60.0)	2 (28.6)	1 (33.3)	1 (33.3)
GS‐positive	7 (22.6)	5 (50.0)	2 (28.6)	1 (33.3)	1 (33.3)
FHM‐positive	6 (19.4)	5 (50.0)	1 (14.3)	1 (33.3)	0 (0.0)
LB/OP	5 (16.1)	3 (30.0)	1 (14.3)	1 (33.3)	–
Miscarriage^e^	2 (28.6)	2 (40.0)	1 (50.0)	0 (0.0)	1 (100.0)

*Note*: Holm test used to determine significance.

Abbreviations: FHM, fetal heart movement; GS, gestational sac; hCG, human chorionic gonadotropin; ICSI, intracytoplasmic sperm injection; LB/OP, live birth or ongoing pregnancy; MII, metaphase II; PB, polar body; PN, pronuclei.

^a^ Steel–Dwass test.

^b^ ICSI cycles using frozen‐thawed spermatozoa, spermatozoa retrieved by testicular sperm extraction, or oocytes that were artificially activated were excluded from the analysis.

^c^ Oocyte with spindle within ooplasm.

^d^ 2PN2PB oocytes cultured until confirmation of blastocyst formation.

^e^ Rate of miscarriage = Patients with miscarriage (*n*)/GS‐positive patients (*n*) × 100.

Using the PPOS, long, or short protocol, the rates of 2PN2PB, 1PN, Multi‐PN, No‐PN, unfertilized, and broken oocytes did not differ based on spindle position (Tables 10, 11, 12). With the GnRH antagonist protocols, the rates of 2PN2PB, 1PN, Multi‐PN, No‐PN, and unfertilized oocytes did not differ based on spindle position (Table 13). The rate of broken oocytes was significantly higher in oocytes with a spindle position of 90° < *θ* ≤ 180° (8/36, 22.2%) as compared with oocytes with a spindle position of 0° ≤ *θ* ≤ 90° (*θ* = 0°, 35/679, 5.2%; 0° < *θ* ≤ 30°, 21/389, 5.4%; 30° < *θ* ≤ 60°, 14/224, 6.3%; and 60° < *θ* ≤ 90°, 5/104, 4.8%, *p* < 0.01, *p* < 0.05, *p* < 0.05, and *p* < 0.05, respectively, Table 13). When we examined embryo development, the rates of blastulation, hCG‐positivity, GS‐positivity, FHM‐positivity, LB/OP, and miscarriage did not differ significantly as a result of spindle positioning after any of the COS protocols (Tables 10, 11, 12, 13).

**TABLE 12:** The relationship between the short protocol, relative position (*θ*) of the meiotic spindle to polar body, and outcomes.

Outcome	*θ* = 0°	0° < *θ* ≤ 30°	30° < *θ* ≤ 60°	60° < *θ* ≤ 90°	90° < *θ* ≤ 180°
Female age, mean years ± SD^a^	35.9 ± 4.2	36.1 ± 4.1	36.3 ± 3.9	36.0 ± 4.1	37.0 ± 3.8
	*n* (%)	*n* (%)	*n* (%)	*n* (%)	*n* (%)
ICSI^b^					
MII oocytes^c^	494	287	184	86	38
2PN2PB oocytes	380 (76.9)	225 (78.4)	139 (75.5)	63 (73.3)	30 (78.9)
1PN oocytes	9 (1.8)	6 (2.1)	7 (3.8)	3 (3.5)	0 (0.0)
Multi‐PN oocytes	20 (4.0)	4 (1.4)	9 (4.9)	2 (2.3)	1 (2.6)
No‐PN oocytes	17 (3.4)	11 (3.8)	8 (4.3)	4 (4.7)	3 (7.9)
Unfertilized oocytes	41 (8.3)	21 (7.3)	9 (4.9)	7 (8.1)	2 (5.3)
Broken oocytes	27 (5.5)	20 (7.0)	12 (6.5)	7 (8.1)	2 (5.3)
Embryo development					
Cultured 2PN2PB^d^	326	192	109	50	22
Blastocysts	207 (63.5)	128 (66.7)	74 (67.9)	37 (74.0)	15 (68.2)
Pregnancy, live birth, and miscarriage					
Blastocyst transfer cycles	76	54	26	14	6
hCG‐positive	25 (32.9)	17 (31.5)	5 (19.2)	5 (35.7)	3 (50.0)
GS‐positive	17 (22.4)	15 (27.8)	4 (15.4)	2 (14.3)	3 (50.0)
FHM‐positive	17 (22.4)	15 (27.8)	4 (15.4)	2 (14.3)	2 (33.3)
LB/OP	15 (19.7)	15 (27.8)	4 (15.4)	2 (14.3)	1 (16.7)
Miscarriage^e^	2 (11.8)	0 (0.0)	0 (0.0)	0 (0.0)	2 (66.7)

*Note*: Holm test used to determine significance.

Abbreviations: FHM, fetal heart movement; GS, gestational sac; hCG, human chorionic gonadotropin; ICSI, intracytoplasmic sperm injection; LB/OP, live birth or ongoing pregnancy; MII, metaphase II; PB, polar body; PN, pronuclei.

^a^ Steel–Dwass test.

^b^ ICSI cycles using frozen‐thawed spermatozoa, spermatozoa retrieved by testicular sperm extraction, or oocytes that were artificially activated were excluded from the analysis.

^c^ Oocyte with spindle within ooplasm.

^d^ 2PN2PB oocytes cultured until confirmation of blastocyst formation.

^e^ Rate of miscarriage = Patients with miscarriage (*n*)/GS‐positive patients (*n*) × 100.

**TABLE 13:** The relationship between the GnRH antagonist protocol, position (*θ*) of the meiotic spindle relative to the polar body, and outcomes.

Outcome	*θ* = 0°	0° < *θ* ≤ 30°	30° < *θ* ≤ 60°	60° < *θ* ≤ 90°	90° < *θ* ≤ 180°
Female age, mean years ± SD^a^	36.7 ± 5.1	37.0 ± 5.1	37.3 ± 5.4	37.7 ± 5.0	37.6 ± 5.5
	*n* (%)	*n* (%)	*n* (%)	*n* (%)	*n* (%)
ICSI^b^					
MII oocytes^c^	679	389	224	104	36
2PN2PB oocytes	521 (76.7)	313 (80.5)	171 (76.3)	80 (76.9)	26 (72.2)
1PN oocytes	15 (2.2)	6 (1.5)	6 (2.7)	5 (4.8)	0 (0.0)
Multi‐PN oocytes	19 (2.8)	15 (3.9)	10 (4.5)	5 (4.8)	2 (5.6)
No‐PN oocytes	25 (3.7)	15 (3.9)	11 (4.9)	4 (3.8)	0 (0.0)
Unfertilized oocytes	64 (9.4)	19 (4.9)	12 (5.4)	5 (4.8)	0 (0.0)
Broken oocytes	35 (5.2)	21 (5.4)	14 (6.3)	5 (4.8)	8 (22.2)**^,§,†,#^
Embryo development					
Cultured 2PN2PB^d^	403	237	139	55	19
Blastocysts	267 (66.3)	156 (65.8)	88 (63.3)	39 (70.9)	12 (63.2)
Pregnancy, live birth, and miscarriage					
Blastocyst transfer cycles	121	47	42	17	3
hCG‐positive	39 (32.2)	14 (29.8)	14 (33.3)	5 (29.4)	0 (0.0)
GS‐positive	28 (23.1)	13 (27.7)	10 (23.8)	2 (11.8)	–
FHM‐positive	25 (20.7)	10 (21.3)	10 (23.8)	2 (11.8)	–
LB/OP	23 (19.0)	9 (19.1)	10 (23.8)	1 (5.9)	–
Miscarriage^e^	5 (17.9)	4 (30.8)	0 (0.0)	1 (50.0)	–

*Note*: Holm test used to determine significance.

Abbreviations: FHM, fetal heart movement; GnRH, gonadotropin‐releasing hormone; GS, gestational sac; hCG, human chorionic gonadotropin; ICSI, intracytoplasmic sperm injection; LB/OP, live birth, or ongoing pregnancy; MII, metaphase II; PB, polar body; PN, pronuclei.

**p < 0.01, each group vs. *θ* = 0°. ^§^ *p* < 0.05, each group vs. 0° < *θ* ≤ 30°. ^†^ *p* < 0.05, each group vs. 30° < *θ* ≤ 60°. ^#^ *p* < 0.05, each group vs. 60° < *θ* ≤ 90°.

^a^ Steel–Dwass test.

^b^ ICSI cycles using frozen‐thawed spermatozoa, spermatozoa retrieved by testicular sperm extraction, or oocytes that were artificially activated were excluded from the analysis.

^c^ Oocyte with spindle within ooplasm.

^d^ 2PN2PB oocytes cultured until confirmation of blastocyst formation.

^e^ Rate of miscarriage = Patients with miscarriage (*n*)/GS‐positive patients (*n*) × 100.

## 4 DISCUSSION

This study demonstrated that after ICSI, the normal fertilization (2PN2PB) rate depends on whether the meiotic spindle is visible in the oocyte and that spindle visibility is affected by the COS protocol employed. However, the location of the spindle within the oocyte cytoplasm (0° ≤ *θ* ≤ 180°) did not affect the rate of normal fertilization after each COS protocol. The blastulation rate differed between oocytes with a visible spindle and oocytes with nonvisible spindle only when the oocytes were retrieved using the GnRH antagonist protocol. Moreover, there were no significant differences in the rates of hCG‐positive, GS‐positive, FHM‐positive, LB/OP, and miscarriage between oocytes with a visible spindle and those with a nonvisible spindle. Accordingly, our results suggest that if fertilized oocytes develop into blastocysts, the visibility of a meiotic spindle in the oocyte does not affect clinical outcomes, such as pregnancy, LB/OP, and miscarriage after ICSI, regardless of which COS protocol was used. Additionally, this study revealed that spindle visibility is related to COS protocol and the hCG‐ICSI interval. Moreover, spindle position, but not spindle visibility, changed with female age and moved away from directly beneath the PB as female age at OPU increased. This is consistent with previous studies, which demonstrated that the spindle position relative to the PB in matured oocytes was affected by in vitro aging and that the first PB degenerates and deviates from the meiotic spindle in aged mammalian oocytes.^24^, ^25^ Spindle deviation in the first PB may indicate low‐quality, aged oocytes.^24^, ^25^ Our data showed that the rate of broken oocytes after ICSI was significantly higher in oocytes with spindle deviation. Hence, our results suggest a decreased tolerance of oocytes with spindle positions of 90° < *θ* ≤ 180° for ICSI.

Our study showed that the clinical outcomes, including the rates of maturation to an MII oocyte, normal fertilization, blastulation, pregnancy, and LB/OP, were superior using the PPOS protocol as compared with other COS protocols. Wang et al.^13^ reported significantly higher rates of MII oocyte and normal fertilization using the PPOS protocol as compared with the GnRH agonist (short, long) and GnRH antagonist protocols. These findings from Wang et al.^13^ are consistent with our results. In contrast, some study findings conflict with ours regarding embryo development after fertilization.^13^, ^26^ Because PPOS is frequently the first choice in our clinic to avoid ovarian hyperstimulation syndrome,^27^ we speculate that this choice may have affected the rates of pregnancy and LB/OP.

This study is the first to our knowledge to focus on the relationship between the visibility and positioning of the meiotic spindle and clinical outcomes after ICSI using different COS protocols. Additionally, our results showed that the rates of visibility of the meiotic spindle increased with time after hCG administration. Although these results are similar to the findings from Kilani et al.,^21^ the rates of spindle visibility after hCG administration peaked at 39.0–40.5 h and declined at 40.5 h in their study. In contrast, our results showed peak visibility 40 h after hCG administration and high visibility continuing 43 h after hCG administration. The rate of spindle visibility did not differ among all hCG‐ICSI intervals using the PPOS protocol. However, the rates of spindle visibility were affected by the hCG‐ICSI interval and were increased with the passage of time using the long, short, and antagonist protocols. Accordingly, our results suggest that the rates of spindle visibility from hCG administration to the completion of ICSI differed among the different COS protocols. In comparisons among COS protocols in this study, the PPOS and short protocols resulted in significantly more oocytes with a visible spindle than the GnRH antagonist protocol. Because the hCG‐ICSI interval was 0.7–1.0 h shorter, this interval may have affected spindle visibility. When we compared oocytes with a visible spindle and oocytes with a nonvisible spindle, the normal fertilization rate was significantly higher in oocytes with a visible spindle, independent of the COS protocol used. This result consisted with previous studies.^2^, ^3^, ^4^

The spindle was directly below the PB (*θ* = 0°) more frequently using the PPOS protocol than with the short or GnRH antagonist protocol. However, we observed significant age differences among female patients undergoing each COS protocol. Notably, the patients who were administered the GnRH antagonist protocol were generally older at OPU as compared with patients given the PPOS, long, or short protocol. Female age was correlated with spindle position, and the spindle moved away from directly beneath the PB as female age increased. Additionally, the association of maternal age with oocyte quality, such as embryo ploidy status, is well established.^28^, ^29^, ^30^ Therefore, this age difference may have impacted ovarian response and the quality of the retrieved oocytes, which in turn impacted spindle position. Generally, female age is related to successful delivery; as female age increases, the fecundability rate decreases and the miscarriage rate increases.^31^, ^32^ Nonetheless, we found that although age at the time of OPU impacted spindle position in the oocyte, it did not affect clinical outcomes such as normal fertilization, embryo development, pregnancy, LB/OP, and miscarriage. The difference in female age between our study groups was at most 1.5 years (range 35.5–37.0 years), however, which may explain why female age at the time of OPU had a minimal impact on clinical outcomes. This interplay is an important area for future investigations.

Our study showed the clinical outcome after ICSI, such as the rates of normal fertilization, blastulation, pregnancy, LB/OP, and miscarriage, did not differ within each COS protocol when the spindle was visible within the cytoplasm, independent of the spindle position. The odds of a spindle located away from the PB were increased in older women. Accordingly, to avoid direct damage to the spindle during ICSI or damage to the spindle due to large deformation of the cytoplasm during ICSI,^33^ confirmation of the position of the meiotic spindle in the oocyte using a spindle‐view system before ICSI is recommended, especially when performing ICSI on oocytes from older women.

This study has some limitations. First, the sample size in the 90° < *θ* ≤ 180°group was small for each blastocyst transfer cycle because fewer MII oocytes were retrieved from the OPU. Second, to reduce the physical and financial burden on patients, we do not perform venipuncture during each ICSI cycle unless the patient's blood data are abnormal. Therefore, we were not able to include blood data, such as anti‐Müllerian hormone and basal hormone levels reflecting each ICSI cycle, in our analyses. Third, this study is a retrospective study, and future prospective analyses are needed to assess the potential impact of these findings on the outcomes after ICSI.

In conclusion, this study demonstrated that the visibility of the meiotic spindle in oocytes impacts the normal fertilization rate, independent of the COS protocol used. Spindle visibility was not associated with female age at OPU, whereas the spindle position was impacted by female age and moved away from directly beneath the PB as female age increased. Our study revealed that spindle position within the ooplasm did not influence the clinical outcomes, including normal fertilization, blastulation, pregnancy, LB/OP, or miscarriage after COS using any of the commonly applied COS protocols.

## FUNDING INFORMATION

This research did not receive any specific grant from funding agencies in the public, commercial, or not‐for‐profit sectors.

## CONFLICT OF INTEREST STATEMENT

The authors declare no conflicts of interest related to this study.

## ETHICS STATEMENT

All procedures were performed in accordance with the ethical standards of the responsible committee on human experimentation (institutional and national) and with the Helsinki Declaration of 1964 and its later amendments. Informed consent was obtained from all patients to be included in the study. This study was approved by the Umeda Fertility Clinic Institutional Review Board (191201).
